# The Epi training kit pilot: an inclusive Spanish-language e-learning approach to epidemiology and data science in Latin America and the Caribbean

**DOI:** 10.3389/fpubh.2026.1745984

**Published:** 2026-05-14

**Authors:** Laura Gómez-Bermeo, José María Velasco-España, Adriana Buitrago-López, Geraldine Gómez-Millán, Zulma M. Cucunubá

**Affiliations:** 1 Institute of Public Health, Pontificia Universidad Javeriana, Bogotá, Colombia; 2 Department of Clinical Epidemiology and Biostatistics, Pontificia Universidad Javeriana, Bogotá, Colombia

**Keywords:** capacity building, data science in public health, gender perspective, infectious disease modeling, localized learning, massive open online course (MOOC), outbreak response training, public health

## Abstract

**Introduction:**

The growing frequency of health emergencies underscores the need to strengthen outbreak-response capacities, particularly in low- and middle-income countries. Barriers such as limited access to training, linguistic exclusion, and gender disparities constrain the adoption of data-science tools in public health. To address these gaps in Latin America and the Caribbean (LAC), the *Epi Training Kit* (EpiTKit), a fully Spanish-language, open-access MOOC, was developed to provide inclusive, gender-aware, and context-sensitive training in infectious-disease epidemiology using open-source software.

**Methods:**

A learner-centered, multimethod design guided the pilot of five learning units delivered via the edX Edge platform. Participants were recruited through a regional training programme. Quantitative data were collected through satisfaction and end-of-course surveys; qualitative data came from two focus groups and open-ended survey responses. Quantitative analysis described demographics, completion, and satisfaction; qualitative analysis used deductive–inductive content analysis to characterize experiences and challenges.

**Results:**

A total of 223 participants from 14 LAC countries took part—Colombia, Dominican Republic, Argentina, Peru, Chile, Brazil, Ecuador, Uruguay, Mexico, Paraguay, Cuba, El Salvador, Panama, and Venezuela. Most were women (66.4%), aged 25–45, and highly educated (54.3% master's, 6.7% PhD), while about 45% were students or held undergraduate degrees. The MOOC achieved a 57.4% completion rate (33.4% among non-mandated learners) and a mean final grade of 83.1. Over 80% rated objectives and resources as effective, and >90% scored units 4–5/5, with highest satisfaction in theoretical modules and lower ratings for R-programming units. Completers rated the course 4.6/5 and 9.3/10 for recommendation; 98% valued the Spanish-language design and 90% recognized its gender-equity focus. Qualitative findings emphasized clarity, inclusiveness, flexibility, and platform usability, but noted time constraints, limited feedback in coding tasks, and occasional English terminology in R scripts/references as remaining barriers.

**Conclusion:**

Spanish-language, gender-aware online training in epidemiology and data science is feasible and well received in LAC. Future iterations should broaden regional datasets and strengthen guided R-learning and feedback mechanisms.

## Introduction

The increasing frequency of health emergencies has underscored the global importance of infectious disease epidemiology and exposed persistent gaps in outbreak response training, particularly in low- and middle-income countries (1–3). Traditional in-person training presents significant accessibility challenges for remote and underserved populations (4). These limitations are further compounded by barriers such as limited access to education, language exclusion, and gender disparities, all of which hinder the adoption of data science tools in public health (5–7).

Among these barriers, linguistic exclusion remains a persistent and often underestimated challenge (8–12). The dominance of English in global health research and education limits access to training, professional development, and international collaboration (8, 9, 11). Language barriers reflect structural inequities that marginalize regions and hinder evidence-based decision-making. In Latin America and the Caribbean, where Spanish and Portuguese predominate, this divide constrains local capacity-building and becomes especially evident during public health emergencies, undermining effective responses (13).

Although non-native English-speaking researchers, trainees, and practitioners often bear the burden of language barriers, addressing linguistic exclusion requires systemic action (8). Promoting multilingual strategies and inclusive training is essential to expand access and reduce inequities. During the COVID-19 pandemic, the WHO addressed this gap by translating key courses into several languages, prioritizing low- and middle-income countries (14, 15). Evidence shows that multilingual training achieves comparable outcomes, highlighting the value of inclusive approaches (10, 11, 16).

The pandemic has accelerated the shift to online education, highlighting Massive Open Online Courses (MOOCs) as scalable, flexible tools for public health training (7, 17–19). While MOOCs provide low-cost, accessible learning via videos, exercises, and forums (20–26), their impact in low- and middle-income countries remains limited by language barriers, digital literacy gaps, unreliable internet, and insufficient local contextualization (7, 27–34).

In Latin America and the Caribbean, MOOC adoption grew after 2015 via platforms like Coursera, edX, and MiríadaX (35). However, participation remains lower than in the U.S. and Europe, mainly due to inequalities in infrastructure and access (36–39). Additionally, most MOOCs are in English, limiting access for Spanish- and Portuguese-speaking learners, despite Spanish being one of the world's most spoken languages (32, 40).

Gender disparities further influence access to education, particularly in STEM fields (41, 42). Although MOOCs have the potential to democratize education, women remain underrepresented, with participation below 25%, despite similar completion rates to men (41, 43, 44). Online courses increase women's STEM participation by providing flexible, accessible options, and programs combining computational skills with real-world applications may help overcome these barriers (45, 46).

Open-source tools expand access to data science training in public health, especially in resource-limited settings, by providing scalable, cost-effective solutions for individual and institutional capacity building. Tools like R, RStudio, and RMarkdown enable hands-on learning and reproducible research, while repositories such as Rpubs and GitHub support global knowledge sharing (47, 48).

To address the intersecting challenges of language, technological access, and gender inequality in Latin America and the Caribbean (LAC), we developed an e-learning strategy tailored to the regional context. This study presents findings from the pilot phase of the Epi Training Kit (EpiTKit), an open-access, Spanish-language initiative aimed at strengthening outbreak response capacities among public health professionals, STEM students, and public health decision-makers in the region. As a MOOC, Epi Training Kit integrates contextualized content, open-source tools, and a gender equity perspective to provide inclusive and regionally relevant training in infectious disease epidemiology.

## Materials and methods

The Epi Training Kit adopts a learner-centered approach, focusing on active engagement and positioning learners at the heart of the educational process (49). This approach fosters active participation, adaptability to individual needs, autonomy, and skill development to create meaningful learning experiences.

We followed Crouch and Broadbent et al. (50), a methodology for pilot planning focused on learning content, training materials, delivery methods, and feedback collection. Based on this, we assessed (i) *course content*: evaluating the relevance, clarity, and quality of the academic content; (ii) *learning objectives*: assessing clarity, achievability, and alignment with the content; (iii) *educational resources*: examining the accessibility, quality, and engagement of materials such as videos, tutorials, and readings; (iv) *platform experience*: evaluating usability, navigation, and technical functionality; (v) *overall feedback*: collecting learners' perceptions, satisfaction, and suggestions for improvement; (vi) *regional context*: assessing if the course reflected the social, cultural, and public health realities of Latin America and the Caribbean; and (vii) *gender perspective*: promoting equity and inclusivity by fostering balanced representation and participation.

Below, we outline the course content, e-learning platform, educational resources, and the implementation of the gender-sensitive approach.

Course content: the course was structured into sequential modules covering (i) the historical foundations of epidemiology, (ii) core epidemiological concepts, and (iii) applied analytical skills using R for data analysis and modeling. This progression was designed to move from conceptual understanding to practical application, enabling participants to build a comprehensive and integrated skill set. The inclusion of historical perspectives supports critical thinking and helps contextualize current epidemiological approaches within broader scientific and public health developments (51).

This structure aligns with established frameworks in epidemiology education, which emphasize the integration of conceptual and applied competencies. In particular, modern training highlights the importance of combining theoretical knowledge with quantitative and computational skills, including mathematical modeling and the use of tools such as R for reproducible data analysis (52–54). Recent literature also underscores the importance of reproducible analytical workflows and the use of open-source tools such as R for simulation and data analysis (55, 56). Therefore, the course reflects current recommendations for comprehensive training in infectious disease epidemiology.

The pilot tested five learning units (Table 1). The proposed duration for the study of each unit was 2 h. For further details see Supplementary Table S1.

**Table 1:** Learning units tested in the pilot of the Epi training kit.

Unit	Description	Educational resources and software tools used
History of epidemics and pandemics	Explores the history of major epidemics and pandemics, from the Plague to COVID-19, with a focus on major epidemics in Latin America and the Caribbean including dengue, yellow fever, cholera, and HIV.	Includes 18 different resources: 10 interactive presentations, 3 explanatory diagrams, 2 expert interview videos, 1 infographic, 1 podcast, and 1 PDF.
Introduction to epidemic theory	Introduces fundamental concepts of epidemic theory including: SIR and SEIR models, measures of incidence and prevalence, and key parameters such as R0, R?, and herd immunity.	Includes 7 different resources: 1 video lecture, 1 explanatory animated video, 4 interactive presentations, and 1 explanatory diagram.
Introduction to R and RStudio	Introduces R and RStudio, an open-source programming language and software environment for epidemiological data analysis, through exploration of the RStudio interface and practical examples involving core R data structures and operators, basic custom function creation, and an introductory overview of data manipulation using the Tidyverse package, providing foundational skills for epidemiological data analysis.	Includes 14 different resources: 6 interactive presentations, 2 explanatory diagrams, 1 infographics, 1 video tutorial, 1 explanatory video, 1 forum, 1 R programming practice, and 1 R challenge using R and RStudio. Key R packages covered include tidyverse, ggplot2, readr, and dplyr.
Data visualization in R with ggplot2	Covers data visualization in R using ggplot2. Through hands-on examples, learners will explore common plot types and learn to enhance them by mapping key components such as data, aesthetics, geometry, scales, facets, and themes. The unit focuses on building a structured foundation for effective visual representation and creating basic plots using ggplo2′s layered syntax.	Includes 13 different resources: 8 video tutorials, 1 forums, 1 interactive presentation, 1 R programming practice, and 2 R challenges using R and RStudio. Key R packages covered: tidyverse, ggplot, dplyr.
Reporting and technical writing in R markdown	Explores R Markdown for automatic, reproducible technical reports by integrating R code with text. By combining Markdown syntax with embedded R code, it streamlines the creation and updating of tables, figures, and analyses without manual formatting, promoting transparent and reproducible data analysis.	Includes 6 different resources: 2 video tutorials, 1 explanatory video, 1 forum, 1 R programming practice, and 1 R challenges using R and RStudio. Key R packages covered: tidyverse, knitr.

e-learning platform: the MOOC was delivered on the edX Edge online platform from Pontificia Universidad Javeriana, an e-learning platform widely recognized in Latin America for its accessibility and open-source framework (57). This platform allows learners to easily access a variety of educational materials, including videos, podcasts, and text, navigate the content intuitively, engage with peers, and monitor their learning progress. It also includes discussion forums that enable participants to interact, share experiences, and exchange ideas, questions, and opinions throughout the course.

Educational resources: the narrative approach positioned participants as “health detectives” uncovering clues to solve public health challenges through themed activities, missions, and characters. A total of 58 educational resources were produced and assembled, including 17 videos, including explanatory animated videos, video lectures, tutorials, and interviews with experts (all with subtitles and transcripts). One podcast, one PDF, two infographics, six animated diagrams, and 21 interactive presentations, along with three discussion forums, four challenges, and three R programming practices all accompanied by explanatory texts to guide learning.

The course also incorporated regionally relevant examples and hands-on exercises grounded in real-world public health contexts. These activities were designed to promote the application of theoretical concepts to practical situations in infectious disease epidemiology. In addition, hands-on programming and modeling tutorials in R were included to enable participants to perform data analysis, thereby strengthening their analytical and problem-solving skills through practical learning (see Table 1).

Gender approach: participant gender balance was intentionally considered and monitored throughout the recruitment and enrollment process to support equitable participation. Moreover, we adopted a gender-sensitive approach by (1) developing a gender-inclusive visual identity, from the course cover to all images presented throughout the modules; (2) highlighting female role models within the course content; (3) ensuring diverse gender representation in multimedia materials, such as videos, images, and podcasts, while avoiding stereotypes; (4) selecting authors and interviewees from varied backgrounds and maintaining gender diversity; (5) creating spaces for gender-awareness discussions among project team members; (6) addressing the underrepresentation of women in the field from the very beginning of the course; and (7) using gender-neutral language, especially in Spanish, where the masculine form is commonly used as generic, avoiding masculine nouns or pronouns as default.

### Participants

A total of 223 participants from 16 countries (14 from LAC) were included in the pilot. They were selected from 592 applicants to Epimodelac 2023, a regular in-person course on Outbreak Analysis and Modeling in Public Health aimed at audiences in Latin America and the Caribbean (58). The pilot participants comprised: (i) 78 top-ranked Epimodelac applicants, who completed the MOOC beforehand; (ii) 128 of the 200 lowest-ranking Epimodelac applicants who were not selected for in-person training; and (iii) 17 trainers and teachers.

In this case, the MOOC was implemented as preparatory material for the in-person Epimodelac training, with the aim of bridging knowledge gaps and consolidating core concepts among participants from diverse academic and professional backgrounds. While Epimodelac focuses on advanced topics in outbreak analysis and mathematical modeling, the MOOC provides foundational training in epidemiology, including historical context, core concepts, and introductory analytical skills using R. This approach enabled participants to establish a common baseline of knowledge prior to engaging with more advanced modeling content.

### Ethical approval and consent to participate

The protocol was approved by the ethics committee of the Faculty of Medicine of the Pontificia Universidad Javeriana, carried out on 09/12/2021 act number 22/2021.

### Data collection

We used a series of methods for data collection, including satisfaction surveys, end-of-course experience surveys and focus groups.

Unit satisfaction survey: both closed- and open-ended questions were conducted within the EdX platform at the end of each unit. Closed-ended items used a Likert scale to measure agreement with statements, while open-ended questions captured their likes and dislikes in natural text language (see Survey Instrument S1).

End-of-course experience survey: survey was sent via email (Microsoft Forms) to both completers and non-completers to explore participants' experiences and perceptions of the MOOC (see Survey Instrument S2).

Demographic variables gathered through the surveys included: age group, gender, country, education level and institutional membership.

Focus groups: focus groups were conducted to gather participants perspectives, ensuring balanced gender representation. Sessions were moderated using structured guiding questions (see Survey Instrument S3) and visual aids such as posters and post-it notes. These were audio-recorded, and transcribed for analysis.

### Data analysis

Quantitative analysis: responses from surveys were summarized using means, standard deviations, and percentages, covering overall experience and Likert-scale feedback on content, learning objectives, resources, study time, and experience with R.

Qualitative analysis: open-ended survey responses and focus group transcripts were analyzed via content analysis, using content analysis that combined deductive and inductive coding. *A priori* categories were defined based on the study objectives, while emergent categories were derived from participants' responses (59, 60). The analytic process included identification of key issues, coding, grouping, critical analysis, moderator input, and synthesis of findings. Analysis was conducted in NVivo 15.

## Results

### Socio-demographic and background characteristics of participants

The pilot of the EpiTKit had 223 participants from 14 countries across Latin America and the Caribbean.

66.4% identified as female, and most participants (54.3%) held a master's degree. The majority were aged between 25 and 45 years, with a concentration between 32 and 38 years and came from various sectors, such as the government, academia, and non-governmental organizations.

Experience with R programming varied, with many beginners (33.6%) and some with no prior experience (18.8%) (see Table 2).

**Table 2:** Participants' demographic characteristics.

Participants total: 223	Women	Men	Total
	148 (66.40%)	75 (33.60%)	
Education level			
PhD	9 (6.00%)	6 (8.00%)	15 (6.70%)
Master	75 (50.60%)	46 (61.30%)	121 (54.30%)
Undergraduate	32 (21.70%)	14 (18.80%)	46 (20.70%)
Other	32 (21.70%)	9 (11.90%)	41 (18.30%)
Country			
Colombia	121 (81.50%)	51 (68.00%)	172 (76.90%)
República Dominicana	7 (4.80%)	4 (5.30%)	11 (5.00%)
Argentina	7 (4.80%)	2 (2.70%)	9 (4.10%)
Perú	5 (3.40%)	4 (5.30%)	9 (4.10%)
Chile	2 (1.40%)	3 (4.00%)	5 (2.30%)
Other LAC country	6 (4.10%)	11 (14.70%)	17 (7.60%)
Age group			
18 to 24 years old	5 (3.30%)	0 (0.00%)	5 (2.20%)
25 to 31 years old	20 (13.60%)	21 (28.00%)	41 (18.40%)
32 to 38 years old	42 (28.30%)	23 (30.60%)	65 (29.10%)
39 to 45 years old	32 (21.70%)	10 (13.40%)	42 (18.90%)
46 to 52 years old	12 (8.10%)	7 (9.20%)	19 (8.50%)
Over 53 years old	3 (2.10%)	2 (2.70%)	5 (2.30%)
NR	34 (22.90%)	12 (16.10%)	46 (20.60%)
Institutional Membership			
Educational	35 (23.60%)	22 (29.50%)	57 (25.60%)
Governmental	19 (12.80%)	11 (14.60%)	30 (13.50%)
NR	92 (62.20%)	38 (50.60%)	130 (58.20%)
NGO	2 (1.40%)	4 (5.30%)	6 (2.70%)
Progress			
Did not start	11 (7.40%)	2 (2.70%)	13 (5.80%)
Less than three units	32 (21.70%)	15 (19.90%)	47 (21.10%)
Completed half of the course	23 (15.50%)	12 (16.10%)	35 (15.70%)
Finished	82 (55.40%)	46 (61.30%)	128 (57.40%)
15.6-7.5,-26243pt Evaluations grade of those who finished [mean (sd)]	83.9 [(14.4)]	81.8 [(17.8)]	83.1[(15.7])
R level			
Advanced	9 (6.00%)	12 (16.00%)	21 (9.40%)
Intermediate	25 (16.90%)	15 (19.90%)	40 (17.90%)
Basic	55 (37.20%)	20 (26.70%)	75 (33.60%)
None	30 (20.30%)	12 (16.00%)	42 (18.80%)
NR	29 (19.60%)	16 (21.40%)	45 (20.30%)

NR, not reported; SD, standard deviation; NGO, non-governmental organization; LAC, Latin America and the Caribbean. LAC countries included: Brasil, Ecuador, Uruguay, México, Paraguay, Cuba, El Salvador, Panamá and Venezuela.

### Quantitative analysis

The MOOC had a 57.4% completion rate, and 73.1% completed at least half of the course (see Table 2). However, the completion rate was not deeply analyzed, as 36% were required to complete the course before attending the in-person event. Out of the 74% who were not mandated to complete the course, the completion rate was 33.4%.

Satisfaction unit surveys: the survey received 630 responses, from 223 participants able to submit up to five responses, one for each unit.

Based on the satisfaction survey (*n* = 630), more than 81% considered objectives and activities effective, 77% felt resources supported learning, and 85% found study time appropriate. Participants reported the highest satisfaction with the theoretical units, while among the programming-based units, those offering more video tutorials, practical activities and challenges received the most positive feedback (see Table 3). In the R-focused units, 74% found instructions clear and 79% said the final exercise reinforced knowledge (see Supplementary Table S2). Overall, over 90% rated units 4–5 on a 5-point scale, with highest scores for early theoretical units and lower ratings for technical ones (see Supplementary Table S3).

**Table 3:** Unit satisfaction percentages.

Unit	Very satisfied	Satisfied	Indifferent	Unsatisfied	Very unsatisfied
History of epidemics and pandemics	84%	14%	0%	1%	0%
Introduction to epidemic theory	79%	19%	1%	1%	0%
Introduction to R and RStudio	60%	30%	8%	2%	0%
Data visualization in R with ggplot2	59%	31%	4%	6%	0%
Reporting and technical writing in R Markdown	41%	42%	9%	8%	0%

A more detailed examination of unit level responses shows variation across specific dimensions of the learning experience. While most units maintained high levels of agreement across indicators, Unit 3 (Introduction to R and RStudio) presented comparatively lower scores in aspects such as content clarity and sufficiency at 75% and usefulness of educational resources at 77% (see Supplementary Table S2). Responses related to the R components within this unit, including clarity of instructions at 74% and applicability through practical exercises at 79%, are also lower compared to other units (see Supplementary Table S2).

End-of-course experience survey: This survey received 105 responses. Of these, 90 were from completers, representing a 71% response rate among those who completed the course, whereas 15 responses came from non-completers.

**Figure 1:**
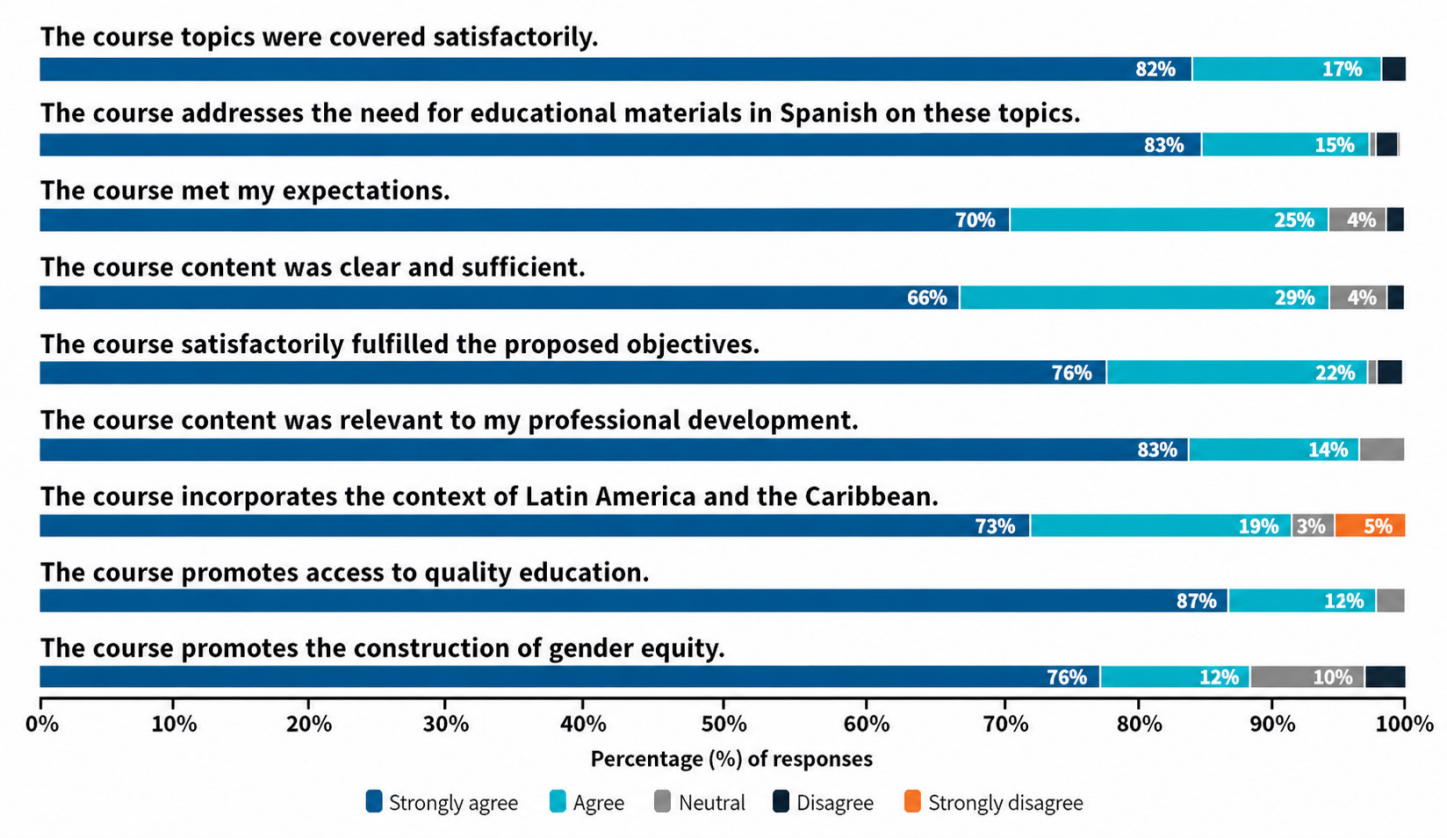
Satisfaction unit survey. Bar chart summarizing participant feedback from the MOOC satisfaction survey via a five-point scale (strongly agree to strongly disagree). It presents responses on course effectiveness, clarity, relevance to professional development, and the integration of regional and gender equity perspectives. The results highlight the MOOC's contribution to reducing educational disparities and promoting inclusion across Latin America and the Caribbean.

In the end-of-course survey (*n* = 90), participants who completed the MOOC rated it 4.6/5 (SD = 0.55) and gave a 9.3/10 recommendation score (SD = 1.2). Overall, 98% agreed that the course addressed the need for Spanish-language materials and promoted quality education. More than 95% found the content clear and sufficient and stated that the course met their expectations. Approximately 92% recognized its regional relevance, and nearly 90% noted its focus on gender equity (see Figure 1). Among non-completers (*n* = 15), 53% cited time constraints, 26% reported technical issues, and 21% mentioned difficulties with the methodology.

At the unit level, satisfaction also varied across modules. The highest proportion of “very satisfied” responses was observed in the theoretical units, including History of epidemics and pandemics at 84% and Introduction to epidemic theory at 79% (Table 3). Lower proportions of “very satisfied” responses were observed in the R related units, particularly Reporting and Technical Writing in R Markdown at 41%, where responses were more distributed across the “satisfied” and “indifferent” categories (see Table 3).

### Qualitative analysis

Two focus groups (*n* = 19) from Colombia, Latin America, and the Caribbean were conducted in person. The first (*n* = 10, 80% female) included participants from Colombian regions. The second (*n* = 9, 40% female) included participants from Brazil, Chile, the Dominican Republic, Ecuador, Mexico, and Peru. In both groups participants aged 25–53 (mainly 32–45) had diverse professional backgrounds. All completed the course, scoring 56–100%, with R proficiency mostly basic (31.6%) or intermediate (42.1%).

Qualitative findings from focus groups and open-ended survey questions reflected an overall positive experience, emphasizing the value of the content, methodology, educational resources, and platform. Participants particularly appreciated the practical exercises that strengthened their R skills and the professional relevance of the content. Reported challenges included language barriers in bibliography and code, limited access to academic papers, and time constraints. The analysis highlighted the need to expand R learning materials and regional representation. Key themes identified in the qualitative analysis included: (1) course content, (2) learning objectives, (3) educational resources, (4) platform experience, (5) regional representation, and (6) gender perspective, which were defined as initial categories based on the pilot objectives. Additional themes emerged from the data, including (7) peer engagement, (8) learning barriers, (9) flexible methodology, (10) R learning experience, (11) interdisciplinary approach, (12) emotional response, and (13) capacity development in public health data science (see Supplementary Table S4).

### Course content

The MOOC pilot content was described as clear, well organized, and relevant. Participants valued the accessible presentation of key concepts, supported by diverse educational resources, and highlighted the practical applicability to their professional work. Participants also reported that the structured, step-by-step progression within the platform enhanced usability and guided the learning process.

*What I liked most about the unit was the theoretical-conceptual approach—it was clear and precise. The most important concepts were covered in a simple way but supported by the literature, which is beneficial for both expert and nonexpert audiences. Additionally, two elements I particularly enjoyed were the podcast and the expert videos, which I believe added extra value*. Survey participant

*I truly liked the didactic way in which the topics were explained, progressing from the simplest to the most complex. They covered concepts that may seem basic, but I believe this is what sets this course apart from others I have taken. Nothing felt like filler, and I finally understood many things I had previously asked about but never got clear answers to. I truly enjoyed the course*. Survey participant

### Learning objectives

Participants praised the course's academic rigor, clear objectives, and relevance to regional public health challenges. They valued learning alongside professionals from diverse disciplines, which promoted collaboration and interdisciplinary. Participants indicated that the MOOC strengthened their knowledge and offered supported its educational use. The alignment between content and objectives enhanced its educational impact. Some suggested adding units on mathematical modeling and advanced analytics to further support capacity building in public health data science. Others highlighted the need to include more data science tools and practical epidemiological applications to connect analysis with public health practice.

*I found the course to have a high technical and academic level, with a well-defined objective that is highly relevant to the region. I appreciate having been selected, and the opportunity to engage with people from diverse disciplines was an added benefit. It provided me with new tools that I hope to apply and share in my work*. Survey participant

*Given the name of the course, I would have expected at least one additional unit, not just about how to use RStudio or ggplot, but about how to calculate prevalence and incidence*. Survey participant

### Educational resources

The educational resources were described as diverse and of good quality, and as supporting engagement. Participants highlighted the combination of readings, infographics, videos, interactive presentations, and practical exercises that facilitated learning and reinforced key concepts. Short videos complemented by written content and downloadable materials supported flexibility and usability.

*The materials are of high quality, innovative, and engaging. The information is clear and to the point, ensuring a concise yet effective message*. Survey participant

*The variety of access options for the materials makes the learning process more manageable and helps maintain attention for longer*. Focus group participant

*I come from Mocoa de Putumayo (a rural area with limited access to the internet and electricity). Interactives don't work very well for me, but I felt comfortable downloading the material and reading it in a traditional way*. Focus group participant

### Platform experience

Participants appreciated the platform's usability, particularly in areas with connectivity challenges. Downloadable materials, interactive content, and a clear visual design enhanced engagement. Despite some internet issues, its lightweight structure enabled smoother access than other platforms, and navigation was considered intuitive and user friendly.

*I'm from Leticia, Amazonas. Regarding the internet: it's not great because we use satellite internet. When it rains, we lose connection, and when there's thunder, we lose power. If it is sunny, the satellite burns out. (...) However, I truly liked the platform that was used because we've tried other platforms before, and they tend to be very heavy. I was worried I would have issues, but I did not*. Focus group participant

*I found it visually appealing, with well-chosen colors, interactive materials, and the option to download content. It offered plenty of support and included diverse resources to accommodate different types of learners*. Survey participant

### Regional representation

Participants recommended broadening the course to include more regional examples from Latin America and the Caribbean. While they valued the rich Colombian content, they noted that incorporating perspectives from other countries would enhance relevance and inclusivity. They also suggested standardizing regional data to strengthen global applicability.

*Standardizing the data in a better way for the international context could be an improvement. Survey participant*

*For me, it felt like a course from Colombia; the accent, the people, the examples, even the experts featured are Colombian. So, it doesn't really feel like a Latin American course*. Focus group participant

### Gender perspective

Participants had mixed perceptions of gender representation in the MOOC. Many praised the visibility of women, particularly female leaders, and valued their inclusion of their contributions. Others suggested making gender representation more visible throughout the course to strengthen inclusivity and contextual relevance, while some felt the current approach was sufficient.

*It's very interesting and wonderful that women have been leading this initiative, and it's great to see how clearly visible their leadership is. (...) I do believe that, in this regard, for instance, the first units highlighted women and significant figures throughout history*. Focus group participant

### Peer engagement

Despite the asynchronous format, participants emphasized that the forums played a crucial role in fostering collaboration, resolving questions, and creating a sense of community. Peer discussions encouraged knowledge exchange and mutual support, enriching the overall learning experience.

*Peer participation in the forum was valuable for successfully conducting the exercises* Survey participant

### Learning barriers

Participants reported challenges such as limited access to bibliographic resources, language barriers, and time constraints. Some struggled with English-only or paywalled materials, which restricted deeper exploration beyond the MOOC. Balancing study with professional and personal duties was also demanding. In technical units, particularly R programming, English commands and functions posed difficulties. Participants suggested adding video tutorials, written code examples, a Q&A section, and an error bank to improve comprehension and support the fully asynchronous learning experience.

*To run the data, there is a lot of terminology in English, even in the videos, and for people like me who are just learning the language, it becomes a barrier*. Focus group participant

*Flexible learning methodology*

The course methodology was valued for its flexibility, enabling participants to balance learning with professional and personal responsibilities. The combination of self-paced learning and structured content allowed participants to engage with the material according to their own schedules.

*I appreciated being able to manage my time. Alternating between readings, videos, and infographics was very helpful in keeping track of the content*. Focus group participant

*It's an interactive course, not tiring, and encourages you to finish it*. Survey participant

### Learning Experience in R Software

Participants valued self-learning and practical exercises for mastering R, noting that hands-on tasks clarified key concepts and supported direct application. Step-by-step video tutorials were praised for clarity and progressive skill building. Those with prior knowledge reinforced their understanding, particularly through practical work. The focus on open-source tools like R was seen as accessible and effective.

*What I liked the most was the self-learning exercise with problem-solving in R, as it enhanced my understanding of the R language*. Survey participant

*Personally, I already had a basic understanding of the R language, and this course helped me better understand certain elements, particularly creating graphics with ggplot. I truly liked the methodology, and I found the pace to be just right*. Survey participant

*I liked that the use of R as a statistical tool is promoted, as it is an open access and free source*. Survey participant

*It's the first time I've been able to do things with R that, although they may seem simple, like a filter, required a lot of effort on my part... I'm truly excited*. Survey participant

*I think learning R is a slow process, so the material could be a bit more explanatory before moving on to the assignments*. Survey participant

*It's very useful to include videos on the use of R, so that the proposed exercises can be developed following the given instructions. I believe that teaching through real demonstrations is a key strategy*. Survey participant

### Interdisciplinary approach

Participants indicated the interdisciplinary approach of the MOOC, which integrated history, public health, mathematics, programming, data science, and epidemiology. This interdisciplinary framework allowed participants to recognize the relevance of diverse fields in addressing complex public health challenges.

*Exploring the connection between history, public health, and epidemiology is enlightening, revealing hidden links between different fields of knowledge. It shows how history can offer valuable insights and help decentralize aspects of the past that inform current public health management*. Focus group participant

### Participants' emotional response

Participants reported emotions ranging from gratitude and enjoyment of the historical sections to frustration with technical and time challenges, especially in the R units. Despite this, they found the course motivating and valuable for professional growth, reinforcing key concepts and inspiring enthusiasm to apply them in their practice.

*I was truly excited. I think it was in the first unit when they gave me an example to ‘calculate the basic reproduction number,' which I had heard about during the pandemic but wasn't very familiar with those types of terms. However, when I did the exercise, everything turned out great, and I thought, “Oh, wow, this is so cool!” That gave me the motivation to keep going and get to this point*. Focus group participant

*It has been a motivational process because it allows you to recall and clarify concepts while taking on new challenges. It has also been quite challenging due to time constraints, as we are at the end of the year and the month, but I enjoy it because I believe it is necessary for the territories to stay updated*. Focus group participant

*From my experience, I felt a bit frustrated because in Units 1 and 2 I did very well, they were about field epidemiology and public health experience, but when I got to Unit 3, I found it very difficult. And I have to confess: there was one unit, I think it was Unit 4, where there were questions in the evaluation and I got 0 out of 3, precisely because I didn't understand. I executed the commands, but they returned an error, and I didn't have anyone I could turn to and say, ‘Look, I'm trying to run the code, but it gives me an error—what can I do?'* Focus group participant

*I also felt a bit uncomfortable with the timing. I think that was the main limitation*. Focus group participant

### Capacity development in data science in public health

Participants indicated a positive experience, emphasizing the knowledge gained in epidemiology and data analysis through tools like R. The MOOC supported their capacity to apply this knowledge in their work and public health decision-making. Developing technical skills in R for data analysis and visualization was highlighted for supporting data-driven approaches in public health.

*It was an enriching experience, as I was able to strengthen my skills in data analysis and visualization through R and ggplot. In my area of work, I am assigned to high-incidence events such as VBD and ARI, and since completing this course, I have started to apply what I learned in my basic and intermediate analytical routines. I hope to continue improving these skills so they can be useful for decision-making. Now, my challenge is to advance in the output of information using ggplot for dissemination spaces such as the COVE, the VBD committee, and epidemiological bulletins*. Survey participant

## Discussion

This study addresses a gap in the literature by describing the pilot implementation of a fully Spanish-language e-learning strategy in infectious disease epidemiology and data science for public health in Latin America and the Caribbean. Guided by learner-centered, community-engaged, and gender-sensitive principles, we collaborated with potential users to identify their educational and technological needs and tailor the strategy to their context. The analysis focuses on piloting the course, including its content, learning objectives, educational resources, platform experience, and the integration of regional and gender perspectives.

The Epi Training Kit was developed to address persistent barriers to outbreak response training, particularly the lack of high-quality, open-access Spanish resources on infectious disease epidemiology, mathematical modeling, and data science. The pilot MOOC received positive feedback for addressing several barriers previously identified in the literature, offering a flexible and scalable training model that helped bridge the digital divide and foster participant diversity. Participants highlighted the course's accessibility, practical relevance, and contextual adaptability. Its broad geographic reach, including remote areas, further demonstrated the effectiveness of this approach.

In addition to its core educational value, the MOOC also played a complementary role within broader training pathways. Specifically, it supports existing in-person training initiatives such as Epimodelac by providing a common baseline of knowledge prior to advanced modeling training. This, in turn, facilitates a more effective learning experience during the in-person course, enabling participants to engage more deeply with complex modeling techniques and applied outbreak analysis.

### Course content

Participants consistently emphasized the value of a rigorous, well-structured course content delivered entirely in Spanish. For many, it was their first exposure to specialized epidemiological content in their native language, underscoring the Epi Training Kit's role in removing a major barrier to comprehension and engagement. The inclusion of technical content, practical exercises, and expert interviews enhanced learning and motivation to apply tools such as R to real-world public health challenges (61, 62), reinforcing the importance of linguistic accessibility in multilingual and low-resource contexts (8, 10).

However, to address the needs of participants who already possess basic knowledge in the field, it is important to broaden the course content by incorporating additional units on data science and mathematical modeling, allowing for a deeper exploration and application of key concepts. For future implementations, adding new units could enhance participants' ability to apply analytical and modeling approaches in public health decision-making.

### Learning objectives

Traditionally, training in infectious disease outbreak analysis has been limited to face-to-face instruction at regional health academies or institutions, often focused on local or region-specific challenges (4). Although this academic approach has addressed some immediate needs, it remains constrained by barriers that limit access for professionals with fewer resources or opportunities to attend such programmes. The Epi Training Kit sought to bridge this gap by offering a flexible, accessible, and regionally relevant learning experience, with objectives aimed at strengthening analytical capacity and applying data-driven approaches to public health decision-making.

Regarding the challenges of learning R programming, the Epi Training Kit seeks to build participants' proficiency in data analysis and statistical software, essential skills for public health practitioners (61, 63). It also aims to encourage a shift toward using R to develop epidemiological thinking and support evidence-based decision-making. By transitioning from traditional menu-driven software such as Excel, EpiInfo, SAS, SPSS, or Stata to open-source programming, the Epi Training Kit promotes a culture of reproducibility, transparency, analytical flexibility, and accessibility through the use of R.

However, while some participants who were introduced to R for the first time through the course successfully achieved the learning objectives, others found it more challenging. For some, the main difficulties were linked to interface complexity and language barriers, often associated with resistance to new technologies (26), whereas others faced broader learning challenges due to limited technical support and opportunities for guided practice. Similar challenges have been identified in analyses of online programming tutorials, which highlight that learners' background knowledge, lack of feedback, and limited opportunities to apply what they learn often hinder progress and confidence in coding (64).

The differences observed across units suggest that participants may experience greater challenges when engaging with more technical and skills based content such as the R modules. While agreement levels remained relatively high for most indicators, the comparatively lower ratings in clarity, resources, and overall satisfaction for Unit 3, together with the declining “very satisfied” responses in later R based units, indicate increased cognitive demands and potential barriers for participants with limited prior programming experience (see Supplementary Tables S2, S3). These findings highlight opportunities to strengthen these units through additional scaffolding, more detailed step by step guidance, and complementary learning resources. At the same time, the consistently high ratings for relevance to professional development underscore the perceived value of these skills, reinforcing the importance of maintaining these components while improving their accessibility in future iterations of the course.

These challenges are also reflected in specific design features of the R units, in the *Introduction to R and RStudio* unit, the lack of video tutorials limited guidance for students who only had access to the code. For the *Data Visualization in R with ggplot2* unit, the absence of feedback on the proposed exercises limited opportunities for students to verify and consolidate their learning. In the Reporting and Technical Writing in R Markdown unit, its position at the end of the course reduced students' ability to fully apply and integrate the skills covered, as many perceived the available time as insufficient to engage thoroughly with the material.

Building on these findings, future implementations would benefit from strengthening learning support resources and providing additional materials to facilitate closer guidance and a smoother transition toward developing programming proficiency and analytical thinking in R. This could include expanding instructional support such as sections addressing common errors and frequently asked questions, as well as allowing more time for completing and engaging with course activities.

### Educational resources

MOOCs offer a unique advantage in reaching broad and diverse audiences, particularly when developed through an interdisciplinary approach. In this course, participants from both STEM and health sectors engaged in a rich exchange of knowledge and perspectives. Diversity extended beyond academic and professional backgrounds to include variations in age, gender, geography, time availability, and access to technology. Addressing these differences required inclusive resources tailored to the needs of such a heterogeneous audience.

In response to this, the course adopted an asynchronous format, allowing participants to access materials at their own pace. While this flexibility supported diverse schedules, it also introduced challenges for engagement and motivation. To mitigate these, the Epi Training Kit employed a learner-centered design that accounted for participants' needs, skills, and contextual challenges.

Research from MOOC pilots and the literature (23, 65–67) shows that engagement improves when courses incorporate diverse resources such as short videos, readings, interactive activities, and discussion forums. This multimodal approach accommodates different learning preferences and supports participants in low-resource settings where video access may be limited (68).

For future implementations, it is important to expand the number of video tutorials available in the R learning units, as code-based materials alone may not be sufficient for all students. This is consistent with the survey results, where lower ratings were observed in aspects related to clarity of content and usefulness of resources in the Introduction to R and RStudio unit, as well as in the evaluation of R instructions and practical exercises (see Supplementary Table S2). For some participants, self-paced video tutorials provide a more accessible and effective way to understand programming logic and troubleshoot errors, particularly when learning independently. As noted in previous studies (64, 69), most students tend to learn by watching videos and practicing through programming exercises at their own pace, and there is evidence that explicit instruction and structured guidance through tutorials can significantly enhance learning outcomes. In this context, expanding audiovisual resources and aligning them with identified gaps in clarity and support could improve comprehension and engagement in these units.

### Platform experience

Learner engagement is shaped by internal assessments that emerge through interactions with the learning platform and peers (70). Interactivity, particularly within MOOC environments, plays a key role in influencing cognitive responses and learning behaviors (71–74). Following Salmon's (66) e-tivity framework, the Epi Training Kit promoted active learning through multiple interactive strategies. Moreover, as several participants highlighted, the platform played a crucial role in supporting their learning experience. For many, challenges such as unstable internet connectivity or power outages were mitigated by the option to download course materials and access them offline, allowing continuity of learning despite technical constraints (66).

### Regional representation

A core goal of the Epi Training Kit is to reflect regional representation across Latin America and the Caribbean, aligning its design and content with the diversity of contexts, experiences, and public health challenges across the region. As suggested by several authors, adopting a context-sensitive design from the outset is essential to ensure that training initiatives effectively respond to regional needs and priorities (27, 29–31, 33, 34). Locally grounded development of open educational resources further enhances their relevance, usability, and impact, particularly by supporting the strengthening of technical capacities in infectious disease modeling and public health data science (28, 36, 75).

However, in this pilot, for the majority of participants from outside Colombia, the content and voices remained largely oriented toward Colombian contexts. For future implementation, efforts should be made to enhance regional relevance by (i) incorporating datasets from other countries in Latin America and the Caribbean, (ii) including experts from diverse national contexts, and (iii) featuring voices with different accents to better reflect the linguistic and cultural diversity of the region.

### Gender perspective

Beyond language and access, the Epi Training Kit also addressed equity in online learning. While MOOCs have been criticized for reinforcing existing inequalities (43, 76–78), this initiative adopted a learner-centered design prioritizing accessibility and inclusion. Key features included downloadable materials, audiovisual transcriptions, and the integration of a gender perspective. Participants valued the presence of diverse voices and female role models, which helped link theoretical content to real-world public health contexts. While opinions varied, with some considering the gender focus sufficient and others feeling a more explicit emphasis was needed, the effort was generally well received.

Furthermore, the Epi Training Kit incorporated efforts to promote gender diversity in STEM, leveraging the flexibility and accessibility of online learning to foster collaboration. Interdisciplinary online courses have been shown to increase the participation of women and underrepresented groups in STEM (43, 79, 80). Accordingly, the Epi Training Kit's integration of online and interdisciplinary approaches provides valuable pathways for advancing gender equity and promoting diversity in STEM.

## Limitations

This pilot study has several limitations. Participants were recruited from applicants to Epimodelac 2023, which may limit generalizability, as they likely had prior interest in epidemiology and modeling. Additionally, 36% of learners were required to complete the MOOC. Although completion rate was not considered a primary outcome in the analysis and results because a proportion of participants were required to complete the course, it may still limit the interpretation of completion as a reliable indicator of learner engagement.

The evaluation relied mainly on self-reported satisfaction and perceptions of learning, without pre- and post-course assessments or longitudinal follow-up to measure objective knowledge gains or real-world application. Qualitative data were collected primarily from course completers, which may underrepresent the experiences of non-completers and those facing greater barriers.

Regarding interactivity, while the MOOC was intentionally designed as a self-paced and scalable learning tool, some participants expressed the need for greater guidance, particularly when engaging with technical components such as R programming. This highlights an opportunity to strengthen the course through the incorporation of additional materials and support tools, while also recognizing that, as is often the case in self-paced courses, some learners may benefit from extra guidance when handling more technical content.

Finally, although designed for the LAC region, the pilot content was perceived as predominantly Colombian, particularly in its case studies, expert voices, and contextual references, which may have limited its perceived regional representativeness and broader contextual relevance across Latin America and the Caribbean.

## Conclusions

The piloting of the Epi Training Kit provided valuable insights for executing effective online training strategies to strengthen public health capacities in Latin America and the Caribbean (LAC). Delivering the course in Spanish and adapting it to the cultural and educational context of LAC, alongside the inclusion of region-specific imagery, voices from local communities, and inclusive language, strengthened learners' sense of recognition and connection. This contextually grounded and context-sensitive approach enhanced the course's relevance and fostered deeper engagement.

Engaging adult learners in asynchronous environments is challenging, particularly for health professionals with demanding schedules. To address this, the Epi Training Kit incorporated diverse resources such as video lectures, infographics, and discussion forums while fostering peer interaction to build community. These elements were crucial to meeting learning goals and promoting the use of data science tools in public health, helping bridge regional educational gaps. However, learning programming in R posed challenges for some participants, highlighting the need for enhanced support and more targeted resources to facilitate proficiency and analytical thinking. Despite these challenges, mastering R remains a key step toward leveraging data science for evidence-based decision-making in public health.

Drawing on the Epi Training Kit experience, future e-learning initiatives must prioritize contextually relevant content, inclusivity, and accessibility to meet learners' diverse needs. Key areas of focus include: (1) providing context-sensitive content with language accessibility and regionally relevant examples; (2) addressing varied social and educational backgrounds through tailored resources; and (3) fostering active community engagement through peer-to-peer knowledge exchange and collaborative activities. Developing multilingual, locally adapted online training that incorporates gender-sensitive approaches is crucial for strengthening regional capacity and ensuring equitable, inclusive access to public health training.

Future iterations of the Epi Training Kit will adopt a structured implementation and evaluation framework to enhance educational impact and regional relevance. Improvements will include systematic incorporation of participant feedback, expansion of regionally representative datasets, integrating additional guided R materials support, and extending time flexibility. Future evaluations will integrate pre- and post-assessments and follow-up surveys to measure knowledge acquisition and application in practice, consolidating the programme as a scalable and regionally adapted strategy for strengthening epidemiological and data science capacity in Latin America and the Caribbean.

## Data Availability

The datasets presented in this article are not readily available because the datasets generated and/or analysed during the current study are not publicly available due to the use of personal and potentially identifiable information from participants and platform users, but are available from the corresponding author on reasonable request. Requests to access the datasets should be directed to zulma.cucunuba@javeriana.edu.co.
