# Supplementary material for: The Epi training kit pilot: an inclusive Spanish-language e-learning approach to epidemiology and data science in Latin America and the Caribbean
Source: Front Public Health. 2026 May 14;14:1745984. doi: 10.3389/fpubh.2026.1745984 (PMC13216479; doi:10.3389/fpubh.2026.1745984)
Supplement: Supplementary file 1 [file Table_1.docx]

**Supplementary Material**

**Table S1. Epi Training Kit Pilot Units and Learning Objectives**

| **Unit** | **Learning Objectives** |
| --- | --- |
| **History of epidemics and pandemics** | - Recognize the major historical milestones in the epidemiology of infectious diseases. - Understand the key concepts related to the origin of epidemics, such as spillover, and distinguish between the terms epidemic, pandemic and endemic. |
| **Introduction to epidemic theory** | - Familiarize yourself with the concepts of epidemic theory (basic SIR and SEIR models). - Understand the differences between incidence and prevalence. - Understand the concepts of basic parameters of epidemic theory. - Reflect on the applications of epidemic theory to some public health problems of infectious diseases. |
| **Introduction to R and RStudio** | - Recognize and make use of R and RStudio. - Know the data types and basic operations in R. - Understand the basic data structures in R, such as vectors and data tables. - Understand the process of creating functions. - Recognize the process of importing, exporting, and transforming databases with Tidyverse. |
| **Data Visualization in R with ggplot2** | - Recognize the functions that make up the ggplot2 package. - Make basic graphs with the ggplot2 structure. |
| **Reporting and Technical Writing in R Markdown** | - Recognize the importance of generating reports in R Markdown. - Learn to use R Markdown in a basic way. |

*Summary of the five learning units of the Epi Training Kit (EpiTKit) pilot program and their corresponding objectives. Describes the expected learning outcomes, covering topics from the history of epidemics to using R for data visualization and technical writing.*

**Table S2. Satisfaction Unit Survey: Questions and Results (Combined Total and Partial Agreement)**

| **Subject** | **Question** | **Unit 1** | **Unit 2** | **Unit 3** | **Unit 4** | **Unit 5** |
| --- | --- | --- | --- | --- | --- | --- |
| **Content** | The content of the unit fulfilled my expectations | 96% | 98% | 81% | 92% | 93% |
|  | The content of the unit was clear and sufficient | 96% | 95% | 75% | 93% | 87% |
|  | The content of the unit was relevant to my professional development. | 96% | 98% | 89% | 94% | 94% |
| **Learning goals** | The learning goals planned at the beginning of the unit were fully achieved | 97% | 98% | 81% | 95% | 93% |
| **Learning activities** | The academic activities were beneficial in achieving the learning objectives. | 96% | 98% | 87% | 93% | 92% |
| **Themes** | The topics of the unit were addressed satisfactorily. | 97% | 98% | 87% | 93% | 94% |
| **Educational Resources** | Resources (videos, infographics, podcasts, documents and texts) facilitated the learning process | 97% | 96% | 77% | 93% | 93% |
| **Study time** | The time invested in completing the unit was adequate to address the content. | 95% | 97% | 85% | 91% | 94% |
| **Experience with R** | The instructions in R were clear and precise, which facilitated the execution of the exercises in a gradual and understandable manner | N/A | N/A | 74% | 91% | 93% |
|  | The application of the acquired knowledge was made possible through the final practical exercise in R | N/A | N/A | 79% | 91% | 92% |

*Closed-ended questions from the unit satisfaction survey and the corresponding results. It shows the percentages of total agreement and partial agreement responses for each question in each of the five course units. Additionally, two Open-ended questions were included: 1) What did you like most about the unit? 2) Do you have any suggestions to improve this unit?*

**Table S3. Rating of overall experience in the MOOC**

| **How would you rate your overall experience in the MOOC?**  **(with 5 being the highest rating and 1 the lowest)** | | | | | |
| --- | --- | --- | --- | --- | --- |
| **Unit** | **5** | **4** | **3** | **2** | **1** |
| History of epidemics and pandemics | 77% | 22% | 1% | 0% | 0% |
| Introduction to epidemic theory | 79% | 19% | 2% | 0% | 0% |
| Introduction to R and RStudio | 48% | 43% | 8% | 0% | 0% |
| Data Visualization in R with ggplot2 | 64% | 32% | 4% | 1% | 0% |
| Reporting and Technical Writing in R Markdown | 67% | 30% | 2% | 0% | 0% |

**Table S4. Categories of Qualitative Analysis**

| **Theme** | **Subtheme** | **Quotes** |
| --- | --- | --- |
| **Course content** | Kind and friendly | *"The content was clear, organized, and to the point. I especially liked that it was concise."* |
|  | Theoretical support |  |
|  | Clear and complete |  |
|  | Organized and Consistent | *"I felt that the knowledge was useful because, in our professional work, these topics are often discussed but rarely applied. This is partly due to barriers to accessing knowledge (...) I think the course is highly useful, challenging, and transformative."* |
|  | Didactic and recursive |  |
|  | Useful examples |  |
|  | Relevant |  |
|  | Progressive learning |  |
| **Learning objectives** | High academic standards. | *"The content aligned with the planned learning objectives, and the concepts were concise and to the point"* |
|  | Applicability |  |
|  | Interdisciplinary collaboration |  |
| **Educational resources** | High quality | *"The interactive explanation using videos and animations of the concepts covered in the unit, along with the combination of animation and lecture-style presentations, is an effective teaching methodology."* |
|  | Downloadable materials |  |
|  | Diversity |  |
|  | Interactive presentations | *"I really appreciated that the explanatory and expert videos were short, allowing me to watch them and then continue with the written content. This kept me engaged and prevented boredom from watching long videos. (…) In some courses, videos are as long as 20 minutes."* |
|  | Forum |  |
|  | Practicals |  |
|  | Short videos |  |
|  | Expert interviews |  |
| **Platform experience** | Intuitive | *“They provide well-structured materials with an intuitive layout for students, highlighting key points effectively. Navigation is a pleasant experience, and the expertise of some instructors is particularly engaging."* |
|  | Usable and accessible |  |
|  | Enjoyable |  |
|  | User-friendly |  |
|  | Engaging design |  |
| **Regional representation** | Regional Representation | *"Just as Colombian cases are included, if this course is meant for a broader audience beyond Colombia, cases from other Latin American countries should also be incorporated."* |
|  | Context Enrichment |  |
| **Gender perspective** | Role model | *"I believe that many see Zulma Cucunubá as a role model, and her representation of women in science is significant. However, this focus did not seem prominent throughout the course."* |
|  | Representation and visibility |  |
|  | Balance |  |
|  | Leadership |  |
|  | Explicit gender approach |  |
| **Peer engagement** | Motivation | *"I really appreciated the sense of camaraderie."* |
|  | Network |  |
|  | Helpful |  |
| **Learning Barriers** | Access to resources | *"There are articles that require payment or other resources to access, but for the course, I think having freely downloadable articles would be ideal."* |
|  | Paywall scientific papers |  |
|  | English proficiency |  |
|  | Knowledge level |  |
|  | Time management |  |
| **Flexible methodology** | Flexibility | *"I really liked the didactic way in which the topics were explained, progressing from the simplest concepts to the more complex ones. They covered things that may seem basic but, in my opinion, set this course apart from others I have taken. Nothing felt like filler content, and I finally understood many things I had asked before but never had explained properly. I truly enjoyed the course."* |
|  | Autonomous work |  |
|  | Resource variety. | *"I feel that it’s a space that supports learning. I felt very comfortable, and everything was explained very clearly. The introduction to practice was excellent. I really enjoyed the course.”* |
|  | Interactive |  |
| **R Learning Experience** | Self-learning | *“The development of the practical exercise allows for a better internalization of the concepts related to R and directly observe their applicability. Attempting to solve these types of exercises enables finding different solutions to a given situation using R. From the outset, the ability to clean and organize databases is extremely useful in the daily work of those involved in data analysis, and optimizing these processes allows for reinvesting time in more analytical rather than operational tasks.”*    *"Regarding R in particular, I think one of the things I missed was the opportunity to have a live session, for example through Zoom, with someone to help answer questions. When you’re learning this for the first time, what you need most is someone to help you get started. The hardest part at the beginning is understanding the logic of how it works. Maybe having a space beyond the forum would help."*    *"It’s very useful to include videos on the use of R, so that the proposed exercises can be developed following the given instructions. I believe that teaching through real demonstrations is a key strategy."* |
|  | Practical exercises |  |
|  | Strengthening data analysis capabilities |  |
|  | Learning tools |  |
|  | Learning methodology |  |
|  | Open source software promotion |  |
| **Interdisciplinary approach** | Cross-disciplinary collaboration | *"This was a pleasant experience, enhanced by interaction with people from other fields."* |
|  | Decentralization. |  |
| **Emotional response** | Acknowledgment | *"It was an enriching experience, spanning from exploring the history of infectious diseases to analyzing data, creating visualizations, and generating reports using R."*    *"I also felt a bit uncomfortable with the timing—I think that was the main limitation. What I was most eager about was the R part, because I wanted to reach this in-person stage (the course) with some foundational knowledge, but trying to understand everything in just a week and a half was really the biggest challenge."* |
|  | Nostalgia |  |
|  | Enthusiasm |  |
|  | Frustration |  |
|  | Motivation |  |
|  | Challenging |  |
|  | Satisfaction |  |
| **Capacity development in public health data science** | Data analysis | *"The course helped me review fundamental epidemiology concepts and provided me with knowledge on data analysis using R."* |

**Survey Instrument S1. Unit Satisfaction Survey**

1. Please indicate your level of agreement with the following statements regarding your knowledge:

| **Survey item** | **Strongly disagree** | **Partially disagree** | **Neutral** | **Partially agree** | **Strongly agree** |
| --- | --- | --- | --- | --- | --- |
| The content of the unit fulfilled my expectations | ☐ | ☐ | ☐ | ☐ | ☐ |
| The content of the unit was clear and sufficient | ☐ | ☐ | ☐ | ☐ | ☐ |
| The content of the unit was relevant to my professional development. | ☐ | ☐ | ☐ | ☐ | ☐ |
| The learning goals planned at the beginning of the unit were fully achieved | ☐ | ☐ | ☐ | ☐ | ☐ |
| The academic activities were beneficial in achieving the learning objectives. | ☐ | ☐ | ☐ | ☐ | ☐ |
| The topics of the unit were addressed satisfactorily. | ☐ | ☐ | ☐ | ☐ | ☐ |
| Resources (videos, infographics, podcasts, documents and texts) facilitated the learning process | ☐ | ☐ | ☐ | ☐ | ☐ |
| The time invested in completing the unit was adequate to address the content. | ☐ | ☐ | ☐ | ☐ | ☐ |
| The instructions in R were clear and precise, which facilitated the execution of the exercises in a gradual and understandable manner | ☐ | ☐ | ☐ | ☐ | ☐ |
| The application of the acquired knowledge was made possible through the final practical exercise in R | ☐ | ☐ | ☐ | ☐ | ☐ |

1. On a scale from 1 (lowest) to 5 (highest), how would you rate your overall experience with the course workshop?
2. What did you like most about this unit?
3. Suggestions for improvement

**Survey Instrument S2. Questions of the End-of-course experience survey**

1. **What is your gender?** Required response. Single choice.
   1. Female
   2. Male
   3. Other
   4. Prefer not to say
2. **Which age group do you belong to?** Required response. Single choice.
   1. 18 to 24 years
   2. 25 to 31 years
   3. 32 to 38 years
   4. 39 to 45 years
   5. 46 to 52 years
   6. Over 53 years
3. **What is your highest level of education?** Required response. Single choice.
4. Bachelor’s degree
5. Specialization certificate
6. Master’s degree
7. Doctorate
8. **What is your academic field of study?** Required response. Single choice.
9. Health sciences
10. STEM fields (Science, Technology, Engineering, and Mathematics)
11. Humanities and social sciences
12. Other
13. **Please rate your level of satisfaction with your experience in the virtual course in Public Health Data Science and Infectious Disease modelling.**

| **Course Component** | **Very dissatisfied** | **Dissatisfied** | **Neutral** | **Satisfied** | **Very satisfied** |
| --- | --- | --- | --- | --- | --- |
| Structure of the units | ☐ | ☐ | ☐ | ☐ | ☐ |
| Quality of educational resources | ☐ | ☐ | ☐ | ☐ | ☐ |
| Duration of the units | ☐ | ☐ | ☐ | ☐ | ☐ |
| Topics selected for the units | ☐ | ☐ | ☐ | ☐ | ☐ |
| Platform quality | ☐ | ☐ | ☐ | ☐ | ☐ |

1. **Please indicate your level of agreement with the following statements about the virtual course in Public Health Data Science and Infectious Disease modelling.**

| **Statement** | **Strongly disagree** | **Partially disagree** | **Neutral** | **Partially agree** | **Strongly agree** |
| --- | --- | --- | --- | --- | --- |
| The course satisfactorily met its stated objectives | ☐ | ☐ | ☐ | ☐ | ☐ |
| The course topics were addressed satisfactorily | ☐ | ☐ | ☐ | ☐ | ☐ |
| The course responds to the need for educational materials in Spanish on infectious disease analysis and modelling | ☐ | ☐ | ☐ | ☐ | ☐ |
| The course met my expectations | ☐ | ☐ | ☐ | ☐ | ☐ |
| The course content was clear and sufficient | ☐ | ☐ | ☐ | ☐ | ☐ |
| The course content was relevant to my professional training | ☐ | ☐ | ☐ | ☐ | ☐ |
| The course considers the Latin America and Caribbean context | ☐ | ☐ | ☐ | ☐ | ☐ |
| The course promotes access to quality education | ☐ | ☐ | ☐ | ☐ | ☐ |
| The course promotes gender equity | ☐ | ☐ | ☐ | ☐ | ☐ |

1. **Please rate your level of satisfaction with each unit of the virtual course in Public Health Data Science and Infectious Disease Modelling.**

| **Unit** | **Very dissatisfied** | **Dissatisfied** | **Neutral** | **Satisfied** | **Very satisfied** |
| --- | --- | --- | --- | --- | --- |
| History of epidemics and pandemics | **☐** | **☐** | **☐** | **☐** | **☐** |
| Introduction to epidemic theory | **☐** | **☐** | **☐** | **☐** | **☐** |
| Introduction to R and RStudio | **☐** | **☐** | **☐** | **☐** | **☐** |
| Data Visualization in R with ggplot2 | **☐** | **☐** | **☐** | **☐** | **☐** |
| Reporting and Technical Writing in R Markdown | **☐** | **☐** | **☐** | **☐** | **☐** |

1. **Which of the following resources from the virtual course in Public Health Data Science and Infectious Disease modelling were useful and supported your learning process?**

Required response. Multiple choice.

1. Videos
2. Infographics
3. Images and diagrams
4. Podcasts
5. Interactive presentations
6. Forums
7. Bibliographic resources
8. R practice exercises
9. PDF documents
10. **How do you rate your overall experience in the virtual course?**

Required response. Rating scale.
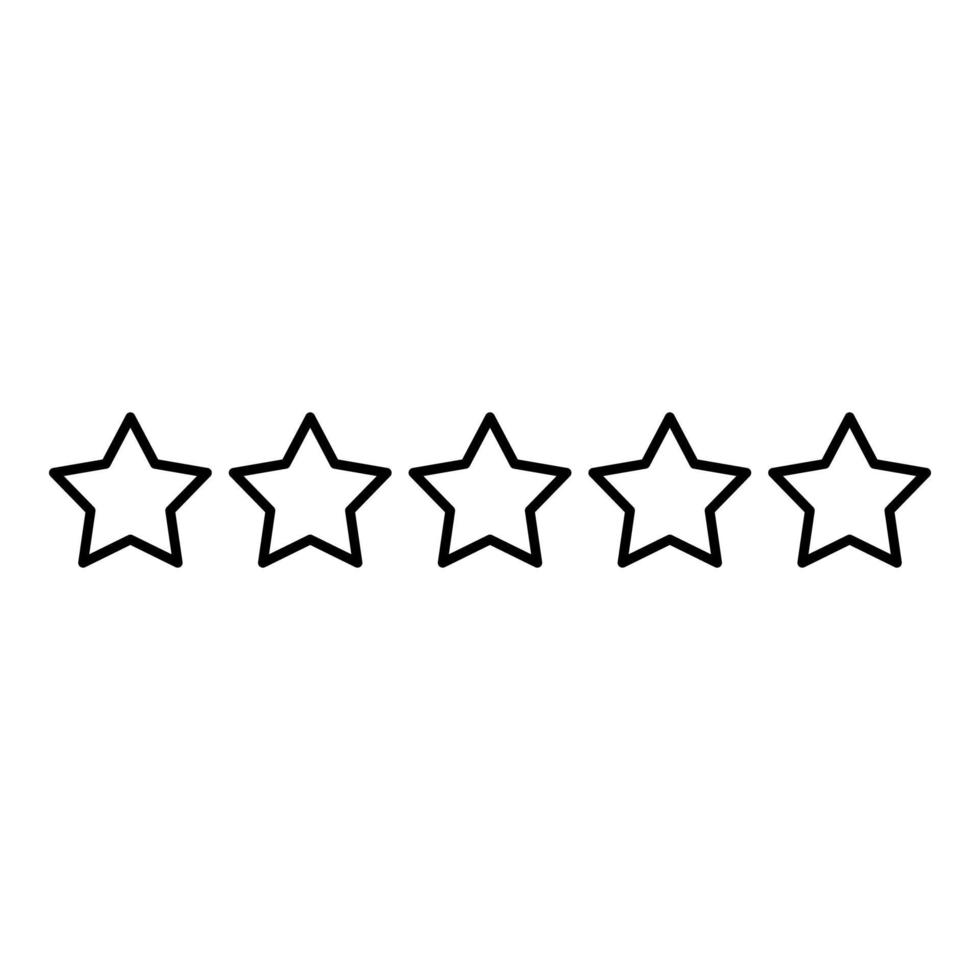


1. **Briefly describe your experience in the virtual course in Public Health Data Science and Infectious Disease modelling.**Multiple-line text.
   Write your response.
2. **What did you like most about the virtual course in Public Health Data Science and Infectious Disease modelling?** Multiple-line text.
    Write your response.
3. **Do you have any suggestions or comments about your experience in the virtual course in Public Health Data Science and Infectious Disease modelling?** Multiple-line text.
    Write your response.
4. **How likely are you to recommend the virtual course in Public Health Data Science and Infectious Disease modelling to others interested in these areas?**Required response. 0 = Not at all likely 10 = Extremely likely
5. **Would you like to participate in the final version of the virtual course in Public Health Data Science and Infectious Disease Modelling?**
   Required response. Single choice.
6. Yes
7. No

**Survey Instrument S3. Guiding Questions for the Focus Group**

**Focus Group Structure**

**EpiKit Pilot Evaluation - Course in Public Health Data Science and Infectious Disease modelling**

**Objective:** Evaluate the experience of the participants in the first pilot of the virtual course on Public Health Data Science and Infectious Disease modelling.

- Explore participants' general satisfaction with the design and structure of the virtual course.
- Obtain detailed perceptions and constructive opinions about the browsing experience of the platform and the course in functional and affective terms in order to identify areas for improvement and strengths that contribute to the final version of the course.
- Evaluate the effectiveness of the didactic resources used, including interactives, diagrams, videos, podcasts, texts and documents.
- Investigate the relevance and clarity of the concepts and topics presented in the course in the context of public health data science and infectious disease modelling.
- Identify possible challenges or difficulties experienced on the platform or with other types of external resources (infrastructure) by the participants during the course and propose suggestions to address them.
- Identify the perceptions of the participants about the inclusion of the gender approach in the pilot of the virtual course.

**Estimated time:** 1 hour

**Date:** Monday, December 4, 4:30pm to 5:30pm

**Place:** Pontificia Universidad Javeriana, Bogotá.

**Number of participants:** 10 in each group, 2 groups in total.

**Table S5. Questions and resources for the Focus Group**

###

| Criteria | Examples | Resources and activities | Guiding Questions |
| --- | --- | --- | --- |
| Effectiveness of educational resources | Explanatory videos, interviews, podcasts, interactive presentations, diagrams, documents | Billboard (2), colored dot stickers Post-its (two colors, for two groups) | Of all the resources that you were able to consult and that were designed to support the learning process in the course, what caught your attention the most? Why?What did you like the most and what do you think can improve your experience (in the use of the resources)?How do you evaluate the effectiveness of the didactic materials provided, such as interactive presentations, videos, podcasts, diagrams, readings, among others?Was there any particular material that you found especially useful or, on the contrary, that needed improvement? |
| Relevance and clarity of content | Module 1 Epidemic and epidemiological theory Units: *History of epidemics and pandemics *Introduction to epidemic theory: Topics | Build a tree of modules, units and themes Post-its | Do you think that the information presented throughout the course to expose and explain the contents was clear and sufficient? Consider: extension, references, clarity, relevanceWas there any aspect of the course that you considered difficult to understand or that needed further clarification? |
| Browsing experience | Course and platform registration, order, intuition in exploring the platform, forum participations, evaluations, downloading resources. |  | How did it feel to navigate the platform throughout the course?What emotions do you associate with that experience?Did you face any specific challenges during the course? If so, how were they addressed or how could they be better addressed in the future? |
| Evaluation of the gender approach | - Make the problem of gender inequality explicit and take a stand.- Take actions in the design, production and exhibition of the content to reduce or not sustain that inequality in the course.-Use of language | Project description of the approach Line of kindness and stickers | Did you feel the course was hostile or friendly?The course includes a gender approach from the presentation. In which we hope that the course presents a friendly environment for everyone. Were all of you able to perceive it? In what way were you able to perceive it?If you had to define the gender approach, how would you define it? Define the approach and see if they agree with that definition.How do you evaluate the inclusion and gender approach in the course content and resources?Can you provide specific examples of how the course incorporated or could improve the incorporation of gender considerations into these topics? Do you consider it necessary?What could improve? |
| Overall course experience |  |  | If you had to briefly define the overall experience of the course, how would you do it?There are always opportunities to improve. If you had to choose some aspect in which we could concentrate our efforts to change/improve something, what would it be? |

### 
